# The Addition of Chinese Herbal Medicines Is Effective as a Prophylactic Treatment Against Dental Diseases for Sjögren’s Syndrome Patients: Insight from Real-World Database

**DOI:** 10.3390/medicina61091537

**Published:** 2025-08-27

**Authors:** Ching-Ya Juan, Wei-Jen Chen, Hanoch Livneh, Ming-Chi Lu, Tzung-Yi Tsai

**Affiliations:** 1 Department of Dentistry, Dalin Tzu Chi Hospital, Buddhist Tzu Chi Medical Foundation, Chiayi 62247, Taiwan; 2 Center of Sports Medicine, Dalin Tzu Chi Hospital, Buddhist Tzu Chi Medical Foundation, Chiayi 62247, Taiwan; 3 School of Post-Baccalaureate Chinese Medicine, Tzu Chi University, Hualien 97004, Taiwan; 4 Graduate Institute of Sports Science, National Taiwan Sport University, Taoyuan 33325, Taiwan; 5 Rehabilitation Counseling Program, Portland State University, Portland, OR 97207-0751, USA; livnehh@pdx.edu; 6 School of Medicine, Tzu Chi University, Hualien 97004, Taiwan; 7 Division of Allergy, Immunology and Rheumatology, Dalin Tzu Chi Hospital, Buddhist Tzu Chi Medical Foundation, Dalin Township, Chiayi 62247, Taiwan; 8 Department of Medical Research, Dalin Tzu Chi Hospital, Buddhist Tzu Chi Medical Foundation, Chiayi 62247, Taiwan; 9 Department of Environmental and Occupational Health, College of Medicine, National Cheng Kung University, Tainan 70428, Taiwan

**Keywords:** Sjögren’s syndrome, dental diseases, Chinese herbal medicines, cohort-based nested case-control study, dental diseases, interdisciplinary collaboration

## Abstract

*Background and Objectives*: Sjögren’s syndrome (SS) is a chronic autoimmune disease that affects the salivary glands and increases the risk of developing dental diseases (DDs). Chinese herbal medicines (CHMs) represent a promising alternative strategy for SS treatment; however, the association between CHMs and DD risk has not been confirmed. In this retrospective, cohort-based, nested case-control study, we explored whether or not combining CHMs with routine treatments for SS can reduce the chance of DDs. *Materials and Methods*: In the beginning, we recruited subjects aged 20–80 years with newly diagnosed SS who were free of DDs between 2001 and 2009 from a nationwide insurance database. We identified DD events that occurred after SS diagnosis until 31 December, 2013. Corresponding controls were randomly selected from the remaining enrollees using a pair-matched approach. We then exploited conditional logistic regression to explore the association between CHM use and subsequent risk of DD development. *Results*: Based on the recruited 586 DD cases and 586 non-DD controls, we noted that adding CHMs to routine SS treatment substantially correlated with a lower risk of developing DDs (adjusted odds ratio = 0.68; 95% confidence interval = 0.52–0.90). Notably, for those receiving CHM treatment for more than 365 days, CHM use greatly reduced DD susceptibility, by 44%. *Conclusions*: Embedding CHMs within routine SS care can prevent subsequent DDs incidence, implying the urgent need for interdisciplinary collaboration and careful treatment planning.

## 1. Introduction

Sjögren’s syndrome (SS) is a common chronic autoimmune disorder with a prevalence in Taiwan of approximately 16.0 per 100,000 persons [1]. Patients with SS would sustain dysregulated immune responses to the salivary and lacrimal glands, thus causing dry mouth and eyes [2,3]. The disease initially manifests in the salivary glands. Autoantigens derived from the salivary glands are recognized by dendritic cells, which triggers the activation of T and B cells and the subsequent release of inflammatory cytokines, leading to immune cell infiltration in the periductal area and the ultimate loss of salivary gland acini [4].

The main functions of saliva include preserving tooth integrity, lubrication, antimicrobial activity, and buffering action [5]. Decreased salivation in SS patients could affect their dental condition, as expected [6,7]. Our previous study showed that patients with SS had a higher prevalence and frequency of dental visits than the control group, as well as a higher incidence of dental caries, pulpitis, gingivitis, periodontitis, oral ulceration, and stomatitis [8]. The presence of pro-inflammatory profiles accompanying SS may induce the impairment of the salivary glands, bacterial colonization, and an inferior gingival index, thus inciting a risk of dental diseases (DDs) [9]. Presently, topical fluoride is commonly prescribed for caries prevention, but the efficacy of decreasing dental caries in SS patients is equivocal in a clinical trial [10]. In addition, pilocarpine hydrochloride, a muscarinic agonist, is commonly used to stimulate saliva secretion and control oral dryness [11]; however, this medication exerts no protective effect on dental caries or periodontitis among them. Given that DDs development in patients with SS is primarily related to the innate immune response, as revealed via miRNA expression profiles [12], complementary treatments might be identified that can be combined with routine medications for DDs prevention among SS patients.

Chinese herbal medicines (CHMs), a specialized form of traditional Chinese medicine, have been extensively used for the therapeutic and prophylactic management of human diseases. The effect of CHMs on the clinical symptoms of patients with SS may be linked to the alleviation of inflammatory mediators [13]. Importantly, the potential bone-protective and anti-inflammatory effects derived from natural herbal extracts have been highlighted in a priori evidence. A late review article noted that the substance of Bei-Mu may greatly modulate levels of nitric oxide (NO) and pro-inflammatory parameters in the serum by inhibiting the mitogen-activated protein kinases (MAPKs)/NF-kappaB (NF-κB) pathway [14]. Another in vitro study demonstrated that the extracted compound Gan-Lu-Yin can significantly restrain the receptor activator of the nuclear factor-kB ligand (RANKL) and NF-κB signaling [15], both of which are known to play an important role in osteoclast differentiation [16], implying the potential benefit for this herbal formula in confronting gene expression during osteoclastogenesis. Despite this research progress, an effective strategy for preventing or delaying the emergence of DDs during SS treatment remains elusive. Given the frequent co-occurrence of SS and DDs, the hypothesis of this nested case-control study was to explore whether the addition of CHMs to conventional SS treatment can minimize the subsequent risk of developing DDs among such groups.

## 2. Methods

### 2.1. Data Source

Taiwan, a modern Chinese country, implemented the National Health Insurance (NHI) program in 1995 to provide coverage for general medical expenses to virtually all of its citizens. As of the present, more than 99% of Taiwan’s population is registered in this program [17]. The data used in this nested case-control study were derived from the NHI research database, which collects all healthcare claims of beneficiaries who have undergone treatment in Taiwanese specialist care, including visits for ambulatory and inpatient care. By and large, this database includes beneficiary information about gender, birth dates, and physician billing claims for inpatient and outpatient visits covered by the NHI, allowing for the identification of all medical consultations and diagnoses. To protect privacy, the identification numbers of persons and healthcare facilities in the datasets have been transformed using end-to-end encryption. The encryption is consistent across all datasets so that the encrypted identification numbers remain unique, making longitudinal follow-ups feasible. All relevant medical diagnoses were coded using the International Classification of Diseases, Ninth Revision, Clinical Modification (ICD-9-CM). As all analytical data were anonymized, the Institutional Review Board of Buddhist Dalin Tzu Chi Hospital confirmed that this study was exempt from full review, along with the need for informed consent in this study (No. B10004021-3). This study was conducted in accordance with the guidelines of the *Declaration of Helsinki*.

### 2.2. Study Cohort

For this retrospective, cohort-based, nested case-control study, we identified subjects aged 20–80 years old with new-onset SS episodes between 2001 and 2009 from the NHI research database. SS diagnosis was confirmed if healthcare-seeking behavior was recorded at least twice in outpatient clinic records within one year or at least once during hospitalization, with an ICD-9-CM code of 710.2. Furthermore, only those with a record of a catastrophic illness certificate (CIC) due to SS were recruited. Under the NHI social program, those with autoimmune illnesses or malignancy can apply for the CIC card to abate the copayment of all medical care. The application of this card must be reviewed by two or more experts, thus endorsing the diagnostic accuracy of the diagnosis. Herein, the date of the first diagnosis of SS was considered the cohort entry date. To adhere to established research procedures, we excluded patients who were followed for less than one year and those with incomplete data (*n* = 218). Additionally, patients diagnosed with an SS episode after DDs were excluded to ensure the appropriate temporal direction of SS and incident DDs (*n* = 4476). The remaining patients comprised the cohort and were followed up until the earliest occurrence of DDs, withdrawal from the NHI program, or the end of 2013, whichever occurred first.

### 2.3. Determination of Patient and Control Groups

**Figure 1:**
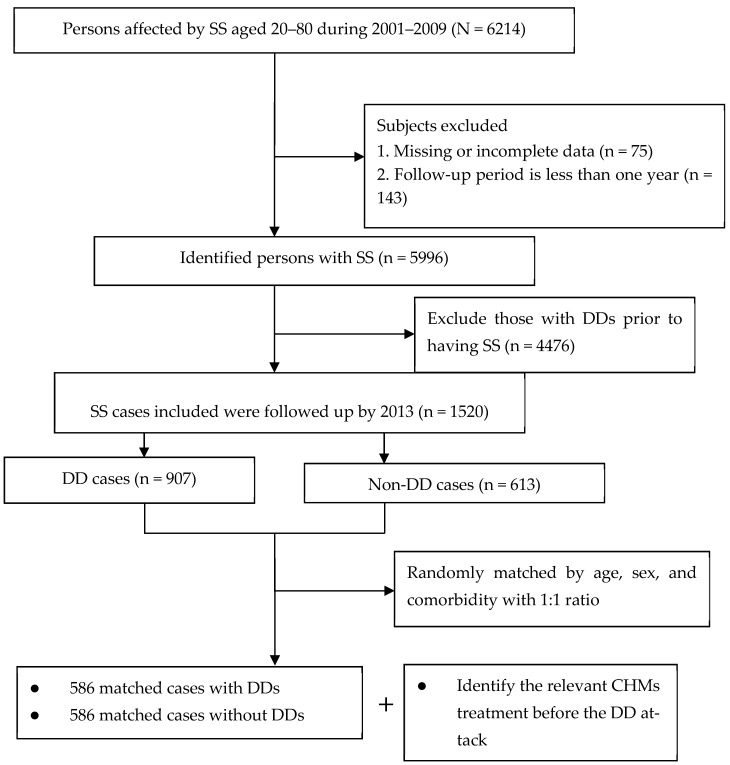
Flowchart of subject selection.

Herein, the primary outcome was the attack of DDs that occurred between 2003 and 2013. Participants were identified as having DDs upon they received at least two outpatient clinic visits in one year or one hospitalization during the study timeframe, such as dental caries (ICD-9 code: 521.0), pulpitis (ICD-9 code: 522.0), gingivitis (ICD-9 codes: 523.0, 523.1, and 523.2), periodontitis (ICD-9 codes: 523.3, 523.4, 523.5, and 523.8), oral ulceration (ICD-9 code: 528.2), and stomatitis (ICD-9 code: 528.0). All of the aforesaid indicators were restricted to those occurring after SS onset. The first medical visit due to DDs was viewed as the index date. Subsequently, each person with a DD was randomly matched to one control without having DDs by age, sex, and comorbidities (Figure 1). An index date was assigned to each of these controls corresponding to the DDs diagnostic date for the study group, thereby ensuring the same observational timeframe for all enrollees.

### 2.4. Identification of CHMs Use

Data on CHMs use were obtained from the medical records of visits to Chinese medicine practitioners that occurred from the cohort entry to the index date. Subjects were designated as CHMs users if they received CHMs treatments due to SS or its symptoms for more than 30 days, and all remaining cases were designated as non-CHMs users. Amongst CHMs users, they were further split into the subgroups according to the sum of CHMs prescription days. This manner allowed us to cautiously appraise the exposure–response association between adjunctive CHMs treatment and the likelihood of DDs development.

### 2.5. Covariate Measures

Based on former research [18,19], the covariates analyzed included sex, age, individual monthly salary, urbanization level in residential areas, and previous medical comorbidities. The premium payment category was employed as a substitute for the monthly salary and was split into three quartile values: lower quartile, median, and upper quartile. Furthermore, the residential districts of study participants were subdivided into three groups according to an established method [20]. It was created based on the following dimensions: population density per square kilometer, the proportion of individuals with a bachelor’s degree or higher, the ratio of elders aged 65 years or older, the percentage of the labor force employed in agriculture, and the number of clinicians per 100,000 inhabitants. Medical comorbidity was defined as a condition diagnosed at least once for an inpatient, or twice for an outpatient, and claims one year preceding the cohort entry. The Charlson–Deyo comorbidity index (CCI), a method for evaluating the number and severity of 17 pre-defined comorbid conditions (range, 1–6), was utilized to assess the impacts [21].

### 2.6. Statistical Modeling

The statistical analytical systems of the SAS software for Windows version 9.4 and SPSS 22.0 constituted the start of this project. Descriptive statistics, including the mean, standard deviation (SD), frequency, and percentage, were applied to analyze the patients’ characteristics at the baseline. Between-group differences were calculated using an independent-sample *t*-test for continuous variables and the chi-square test or Fisher’s exact test for categorical variables, as appropriate. Conditional logistic regression, with DDs as the major dependent variable and CHMs exposure, age, sex, individual monthly salary, residential urbanization level, and comorbidities captured at the baseline as independent variables, was executed to estimate odds ratios (ORs) and 95% confidence intervals (CIs). We then determined crude ORs based on a simple model including only CHMs use, as well as adjusted ORs for a full model, including all participant background variables. In all statistical tests, a *p*-value of 0.05 or lower was deemed significant.

## 3. Results

Of the recruited study cohort, the persons afflicted with DDs and without DDs contributed data on 586 patients each. The corresponding sociodemographic and clinical features are shown in Table 1. The mean age was 59.9 years (SD, 13.6), and nearly 87% were female. The majority of enrollees had a median monthly income level (55.2%) and tended to live in more urbanized areas (64.9%). Pertinent characteristics, such as age, sex, monthly income, residential area, and comorbidities, did not differ significantly between the DD and control groups.

Within the study period, 21.3% of DDs cases and 28.3% of non-DDs cases used CHMs remedies. From the multivariable analyses of CHMs use and DDs risk, we observed that CHMs users exhibited a lower risk of being diagnosed with DDs in comparison with the comparators (adjusted ORs, 0.68; 95% CI, 0.52–0.90). Notably, those receiving CHMs for more than 365 days possessed the most striking reduction in DDs, with the ORs of 0.56 (95% CI, 0.35–0.92). This finding unfolds an inverse exposure–response association between the duration of CHMs use and the probability of having DDs (Table 2). On top of that, a greater benefit in the prevention of DDs was observed among female subjects, especially for those aged ≤50 years, with an adjusted OR of 0.45 (95% CI, 0.25–0.82) (Table 3).

**Figure 2:**
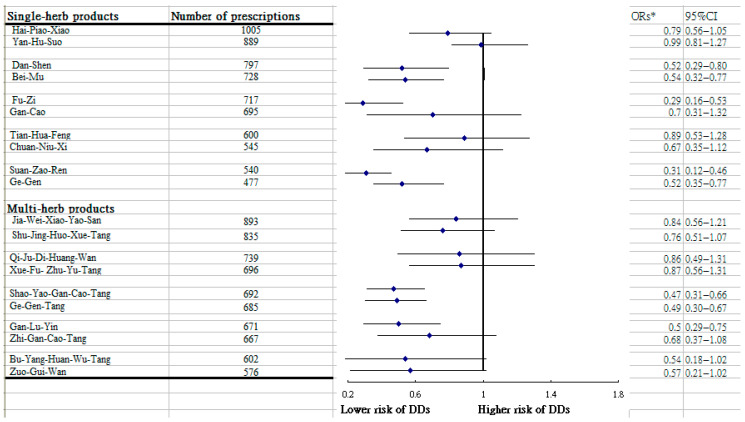
DDs chance calculated via multivariable conditional logistic regression among the most used single-herb and multi-herb formulas for SS patients. * Model adjusted for residential area, age, sex, monthly income, and CCI.

Of the most common CHMs formulas for treating SS, several were correlated with a lower likelihood of DDs, which included Shao-Yao-Gan-Cao-Tang, Ge-Gen-Tang, Dan-Shen, Fu-Zi, Bei-Mu, Suan-Zao-Ren, Ge-Gen, and Gan-Lu-Yin (Figure 2).

## 4. Discussion

SS is a chronic inflammatory disease that frequently affects the functions of the lacrimal and salivary glands. Despite excellent oral hygiene, individuals with SS still have an elevated risk of developing DDs because of the excessive activation of innate immune responses [3,7]. Therefore, for persons living with SS, oral symptoms must be effectively managed to lessen further negative impacts the quality of life. The results of this study showed that integrating CHMs into routine care for DDs would significantly decrease the likelihood of DDs development. Specifically, we observed a 44% decrease in the risk of developing DDs among patients using CHMs for more than 365 days. Although direct comparisons with matched relatives of target patients are scarce, the positive relationship between CHMs use and reduced DD risk observed in this study confirms previous studies and bolsters a growing body of evidence on this issue [15,22]. The ability of CHMs to prevent DDs may be related to the functions of relevant phytochemicals purified from these herbs, which include the regulation of inflammatory responses and the promotion of bone mineral density, both of which are involved in the development of DDs [12,23,24]. Additionally, the current study indicated that younger females benefited more from CHMs treatment in lessening DDs risk. This may be explained by the knockdown of hormone releases, especially the release of estrogen, which can strongly suppress the activation of inflammatory mediators, such as interleukin (IL)-1β, IL-6, and tumor necrosis factor-α (TNF-α) [25]. This phenomenon has been justified in different experimental models exploring the relationship between depression-like behaviors and estrogen deficiency [26].

The master stroke of this study is its exploration of the CHMs products that may potentially benefit from a reduction in developing DDs among patients with SS. Of the commonly used multi-herb products, we found that a lower risk of DDs among those taking Gan-Lu-Yin and Shao-Yao-Gan-Cao-Tang. Compatible with the previous report illustrating that Gan-Lu-Yin is often prescribed to cure the SS symptoms [27], our study further suggested that this herb was beneficial in minimizing the subsequent risk of DDs among such a group. One recent animal study revealed that rats administered an extract of Gan-Lu-Yin (60 mg/kg) daily for 20 days experienced a reduction in circulating levels of bone resorption markers by approximately 27% compared to the control group with distilled water, thereby abating the predisposition of periodontitis-associated bone destruction [15]. The observed therapeutic benefit of Shao-Yao-Gan-Cao-Tang may be attributed to its proven anti-inflammatory effect. Experiments in a rodent model showed that this formula can significantly decrease levels of plasma cytokines, driven by an aberrant inflammation response, by inhibiting the TLR4/NF-κB pathway [28]. The aberrant activation of the TLR4/NF-κB signal would contribute to secretions of inflammatory cytokines, such as TNF-α, IL-1, and IL-6; all of these mediators are known to deeply involve the development of DDs [12,16,23]. Likewise, we noted that some commonly prescribed CHMs formulas targeting SS, such as Ge-Gen-Tang and Ge-Gen, were both linked to reduced odds for DDs. According to previous research, compared to untreated controls, mice fed with pueraria lobata, a major ingredient from these herbs, experienced lower inflammatory-induced cytokine levels through the down-regulation of NF-κB/p65 activation and the inhibition of NF-kB activity [29]. Anti-inflammatory effects from these polyherbal formulations may point to the possible reasons why these herbs work in persons with SS.

Among other single-herb products used to treat those afflicted with SS, we found that uses of Fu-Zi and Dan-Shen achieve a benefit in the prevention of DDs as well. Radix Codonopsis (Dan-Shen), derived from the dried root of plants in the Campanulaceae family, is a widely prescribed herb, thanks to its admired anti-inflammation and anti-tumor properties [30]. Several modern reports based on human and animal models documented that this formula can significantly ameliorate levels of inflammatory milieu in the plasma, like IL-17A and IL-6, as well as TNF-α [31,32]. Meanwhile, one recent in vivo study has shown that this herb exerted an anti-osteoporosis effect by suppressing the activations of TRAF6 and NFATc1, both of which played imperative roles in osteoclast differentiation during the advancement of periodontal diseases [33,34]. In addition, the use of Fu-Zi was proven to exert a therapeutic effect against the attack of DDs. Utilizing a rodent model, scholars noted that Fu-Zi can markedly promote the proliferation rate of mouse bone marrow mesenchymal stem cells (BMSC) at up to 122.24% when compared to untreated cells [35]. Presently, the BMSC-derived exosomes have been recognized to effectively improve the migration, proliferation, and osteogenic differentiation of human periodontal ligament stem cells [36]. These underlying mechanisms may, therefore, account for the advantages of Fu-Zi and Dan-Shen reported in this study.

Other single-herb products shown to be effective in reducing DDs risk comprised the formulas of Suan-Zao-Ren and Bei-Mu. Mounting pharmacological studies reported that Suan-Zao-Ren decoction possessed the therapeutic efficacy of anti-inflammation, together with an antibacterial impact through the regulation of the TLR4/MyD88/NF-κB pathway [37], possibly inhibiting inflammation throughout the body. Additionally, after administrating Bei-Mu, our study revealed that the association of SS with a subsequent likelihood of DDs would be reversed, with a risk reduction of nearly 50%. In traditional Chinese medicine, Bei-Mu is frequently employed to relieve pain. Its renowned biological activities, especially its anti-inflammatory and anti-aging activities, are emerging as new research hotspots. Indeed, Bei-Mu may regulate the levels of inflammatory precursor substances by inhibiting the mitogen-activated protein kinase/NF-κB pathway [14]. Not only is it the fulcrum of the inflammatory response, but this inflammation-associated cascade is also highly correlated with the induction of matrix metalloproteinase [38], which eventually aggravated degradations of connective tissue and the underlying alveolar bone shown in earlier human and animal experiments [23,39].

Despite its important public health implications, the present study raises some questions that must be addressed. First, while applying secondary healthcare databases, errors in the coding process may be inevitable. To minimize this drawback, we only included individuals with new-onset SS or DDs, and only after they had at least two outpatient visits, reporting consistent diagnoses or at least one inpatient admission. Furthermore, because the coding system and data availability were similar for the two groups, any misclassification bias would likely have been non-differential, thus attenuating the real effect estimates to produce bias toward the null hypothesis. The second limitation relates to the neglect of other predictors of DDs, including smoking status, physical activity, and dietary intake, which may affect the findings to some extent. However, in this study, the use of frequency-matching approach, together with the multivariable analysis performed, should partially control for this concern. Relatedly, given the magnitude and statistical significance of the observed effect in this large-scale survey, these limitations are unlikely to have affected our findings. Third, a surveillance bias may have occurred, as the earlier experience of traditional Chinese medicine usage before cohort entry may influence the following incentive to adopt the treatment of CHMs. To handle this concern, we calculated the individual frequency of CHMs use one year preceding cohort entry, determined by registered practitioners of traditional Chinese medicine. The mean frequencies of ambulatory care for CHMs for the two groups were 1.69 and 1.84, respectively. The reanalysis revealed that use of CHMs still had a significant benefit in abating DDs attack after adjusting for this indicator in the multivariable analysis, with an overall adjusted ORs of 0.71 (95% CI = 0.60–0.82). Lastly, despite the substantial benefit of CHMs in reducing the risk of DDs, it must be acknowledged that participants were not randomly categorized into the study’s two groups. Therefore, future studies, on the basis of a larger randomly assigned cohort of SS patients, should be given top priority to delve into the potential mechanisms through which CHMs prevent DDs’ onset. Despite the above methodological concerns, our study has provided the following contributions. All analyses corresponded to population-based data, consisting of both men and women, which can be comprehensive in assessing the benefit of CHMs on the prevention of DDs among persons with SS. Moreover, the application of a nested case-control study design is a promising alternative to cohort analysis that takes into account the presence of time-dependent exposure, such as the interventions applied via CHMs treatments. Hence, our study could be viewed as comparable in its efficiency to randomized controlled trials in reflecting and analyzing real-world data.

## 5. Conclusions

Our final analysis indicated that individuals who used CHMs treatment in addition to conventional therapy for SS would benefit from a reduction in DDs risk. The observed therapeutic effect appeared to be dose-dependent, with longer CHMs treatment greatly reducing the likelihood of DDs. In addition to providing insights into the therapeutic impact of CHMs on the prevention of DDs, this study paves the way for further in vivo studies on the mechanisms of specific herbal prescriptions in the management of oral conditions. In terms of clinical implications, our findings suggest that rheumatologists should monitor oral health on a regular basis. At the same time, they should inform SS patients about the potential risk of suffering from DDs and encourage them to vigilantly monitor for relevant symptoms by themselves. In the long run, adequate instruction concerning oral hygiene administered via an interdisciplinary collaboration has become increasingly essential to provide comprehensive and effective care for SS patients.

## Figures and Tables

**Table 1:** Demographic data and selected comorbidities among participants.

Variables	Numbers (%)	DDs Cases	Non-DDs Cases	*p*
		*n* = 586 (%)	*n* = 586 (%)	
Age (years)				0.06
≤50	291 (24.8)	160 (27.3)	131 (22.4)	
>50	881 (75.2)	426 (72.7)	455 (77.6)	
Mean	59.9 (13.6)	59.1 (13.7)	60.6 (13.5)	0.07
Sex				0.90
Male	394 (33.6)	196 (33.4)	198 (33.8)	
Female	778 (66.4)	390 (66.6)	388 (66.2)	
Monthly income				0.16
25th percentile	498 (42.5)	249 (42.5)	249 (42.5)	
50th percentile	646 (55.2)	318 (54.3)	328 (56.0)	
75th percentile	27 (2.3)	19 (3.2)	9 (1.5)	
Residential area				0.26
Urban	560 (47.7)	275 (46.9)	285 (48.6)	
Suburban	201 (17.1)	111 (18.9)	90 (15.4)	
Rural	411 (35.1)	200 (34.2)	211 (36.0)	
CCI	5.9 (9.8)	6.4 (10.3)	5.5 (9.3)	0.13

Abbreviations: SD, standard deviation; CCI, Charlson–Deyo Comorbidity Index.

**Table 2:** Relationship between use of CHMs and risk of DDs onset during the study timeframe.

CHMs Exposure	Patients				Crude ORs (95% CI)	Adjusted ORs * (95% CI)
	DDs Cases *n* = 586		Non-DDs Cases *n* = 586			
Non-CHMs users	461	78.7	420	71.7	1	1
CHMs users	125	21.3	166	28.3	0.69 (0.53–0.89)	0.68 (0.52–0.90)
Group 1 (31 days–364 days)	96	16.4	116	19.8	0.72 (0.54–0.98)	0.73 (0.54–0.98)
Group 2 (365 days or longer)	29	4.9	50	8.5	0.57 (0.36–0.94)	0.56 (0.35–0.92)

* Adjusted for potential confounders including age, sex, residential area, monthly income, and CCI. Abbreviations: CHMs, Chinese herbal medicines; ORs, odds ratios; CI, confidence interval; CCI, Charlson–Deyo Comorbidity Index.

**Table 3:** Age- and sex-specific DD risk among SS patients in relation to use of CHM treatment.

	Crude ORs (95% CI)	Adjusted ORs * (95% CI)
Female		
≤50	0.44 (023–0.80)	0.45 (0.25–0.82)
>50	0.65 (0.20–0.95)	0.64 (0.20–0.98)
Male		
≤50	0.72 (0.49–1.20)	0.71 (0.48–1.18)
>50	0.94 (0.52–1.64)	0.93 (0.53–1.62)

* Model adjusted for residential area, monthly income, and CCI.

## Data Availability

The original contributions presented in the study are included in the article, further inquiries can be directed to the corresponding authors.
